# Rapidly Shifting Concepts Regarding Androgens and Prostate Cancer

**DOI:** 10.1100/tsw.2009.80

**Published:** 2009-07-27

**Authors:** Abraham Morgentaler

**Affiliations:** Department of Surgery, Beth Israel Deaconess Medical Center, Harvard Medical School, Boston, USA

**Keywords:** testosterone, prostate cancer, risk, testosterone replacement therapy, hypogonadism

## Abstract

There has been a recent dramatic shift in our understanding of the relationship between androgens and prostate cancer (PCa). Whereas for several decades it had been assumed that higher serum testosterone (T) concentrations would lead to ever-greater PCa growth, current literature indicates that PCa growth is unaffected by changes in serum T throughout most of the naturally occurring range. A Saturation Model has been proposed to explain how prostate tissue can be exquisitely sensitive to changes in serum T at the very low end of the concentration range, but appears indifferent to such changes above the near-castrate range. This has special applicability to T-deficient men, since this means that T therapy may not be nearly as risky as once assumed. Indeed, one of the more interesting changes over the last several years has been the growing acceptance of the use of T therapy in men with a prior history of PCa, with early data indicating minimal risk of cancer recurrence or progression. Provocative new evidence suggests that it is not high serum T that is problematic for PCa, but *low* serum T that is associated with worrisome cancer features and outcomes, such as high Gleason score, advanced stage of presentation, and increased risk of biochemical recurrence after surgery. It will be interesting to see what changes will occur in this rapidly changing field over the next several years.

## INTRODUCTION

In 1941, Huggins and coworkers established a critical relationship between androgens and prostate cancer (PCa) by reporting that the severe reduction in serum testosterone (T) by castration or estrogen treatment caused PCa regression[1,2]. They also reported that T administration caused PCa progression, an assertion that began the decades-long concern that T therapy in hypogonadal men may cause PCa, or may convert an existing, quiescent cancer into a clinically important one.

The belief that higher T leads to greater PCa growth has been considered axiomatic for several decades[3]. However, it is only recently that this concern has been carefully examined[4,5], prompted by increasing interest in the use and benefits of T therapy for T-deficient men. A number of recent reviews has brought into serious question the traditional view regarding the relationship of androgens and PCa, particularly since serum T levels appear unrelated to PCa risk[6,7]. This shift in our understanding of the relationship between androgens and PCa has important clinical ramifications, especially for the use of T therapy in hypogonadal men.

The arguments supporting the concept that T caused PCa growth were multiple: androgen deprivation caused PCa to regress, cessation of luteinizing hormone releasing hormone (LHRH) treatment was associated with a substantial rise in prostate specific antigen (PSA) as T increased into the normal range, and the occasional new diagnosis of PCa in a patient receiving T replacement therapy appeared to confirm the danger of T for men with occult PCa[8]. No wonder, then, that the relationship between T and PCa has been termed as “fuel for a fire” or “food for a hungry tumor”[3].

This concern regarding PCa and T led to the lifetime prohibition against T therapy for any man diagnosed with PCa, regardless of disease status. It has even been suggested that men at higher risk for development of PCa, such as those with a family history of PCa, be excluded from T therapy trials due to the concern that higher T will cause growth of occult cancer in these men[9].

Although we are still without large, long-term controlled studies of T therapy in hypogonadal men, a substantial body of evidence strongly suggests that these traditional fears are unfounded, and that the relationship of T to PCa is significantly different than what has been assumed for many decades. Indeed, one of the more interesting and controversial issues at present is whether T therapy may be safely offered to men with PCa, a concept that would have been universally condemned just a few years ago. This review summarizes our current understanding of T and PCa.

## Revisiting the Original Model of Androgens and PCa

The original view that PCa is androgen dependent stems from the early experiments by Huggins and others in which men with metastatic disease underwent castration or treatment with estrogen to lower serum T[1,2]. Clinical benefits were noted, as well as a reduction in the blood test acid phosphatase, which had shown some utility in the diagnosis of metastatic, but not localized, PCa. This beneficial response seen with severe reduction of serum T to castrate levels has withstood the test of time, and remains a standard form of therapy for men with advanced PCa to this day. In addition, it was reported that administration of T via daily injections of T propionate was associated with an increase in acid phosphatase, and clinical progression or even death in many of these castrated men[10,11]. Clearly, withdrawal of T and its repletion leads to powerful effects on PCa and supports the concept that PCa is androgen dependent.

In 1967, Prout and Brewer noted clinical progression or death in five of 10 men who had been previously castrated and now presented with recurrent disease[10]. In 1981, Fowler and Whitmore reported that 45 of 52 men with bony metastases from PCa treated with T administration at Memorial Sloan Kettering Cancer Center developed an “unfavorable response”, most within 30 days[11]. These reports, combined with the original cases presented by Huggins and coworkers[1,2], formed the basis for the long-standing view that giving T to a man with PCa was like “feeding a hungry tumor”, or “pouring gasoline on a fire”.

This view characterized medical opinion for many decades, despite an accumulation of studies and observations that provided evidence to the contrary. For instance, PCa became highly prevalent and caused increasing mortality as men aged, a time when serum T was known to be in decline. Conversely, PCa was almost unknown in men in their twenties, a time when serum T was known to be at its lifetime peak.

In retrospect, the early historical data central to the concept that more T leads to greater PCa growth also provided key clues to a more sophisticated view of the relationship of T and PCa. In addition to the rapid progression noted by Prout and Brewer in a group of 10 men with recurrent disease after castration, these authors also noted no progression in another group of 26 men who received daily T administration[10]. This group consisted of 20 men who were intact (not castrated) and six men who had just recently undergone castration. Fowler and Whitmore noted that all but four of the men in their study had already been treated with castration or estrogen. Among the four untreated men, three of them did not experience any obvious T-related progression, receiving daily injections of T for 52, 55, and 310 days, respectively[11].

The lack of obvious clinical progression in these untreated men who received T provided a remarkable alternative possibility: perhaps there was a limit to the ability of androgen to stimulate PCa growth. This would explain why PCa appeared to regress or grow with changes in serum T when it was brought into or out of the castrate range, but why intact men with naturally occurring T concentrations did not seem to experience PCa progression with additional T. This concept, now known as the Saturation Model[5,7,12], appears to resolve the known evidence regarding effects on PCa of castration, T repletion in castrated men, effects of T therapy on PSA in hypogonadal men, and impact of serum T on risks of PCa in untreated men[6].

## THE SATURATION MODEL

Whereas the traditional view of T and PCa suggested a continuous relationship between serum T and PCa growth ("More T more PCa growth"), current evidence suggests a bimodal relationship[12]. At very low serum T concentrations, there appears to be exquisite sensitivity of PCa growth to changes in available androgen. However, at some concentration, termed the Saturation Point, prostate tissue appears to become indifferent to changes in serum T. This model applies not only in humans, but also in animal studies and PCa cell lines[12]. Exposure to increasing concentrations of androgen causes greater cell growth, but this growth rate plateaus, and once this saturation point is reached, even logarithmic increases in androgen concentrations fail to elicit greater growth[12].

In humans, healthy men exposed to supraphysiologic doses of T for periods up to 40 weeks failed to demonstrate a rise in PSA or prostate volume[13,14]. Trials of T therapy in hypogonadal men demonstrated a PSA increase of approximately 15% at one year, a value that is significantly greater than baseline in most studies, but not greater than the increase seen in placebo-treated men[12].

At least two mechanisms appear to contribute to the saturation of prostate tissue with regards to androgen. The first is that there is a finite ability of the androgen receptor (AR) to bind androgen[12,15,16]. Since it is the bound complex of androgen-AR that binds to nuclear DNA to exert the proliferative effects of androgen, the point of maximal androgen-AR binding is of considerable importance. In rats, maximal binding appears to occur at 2–3 n*M* (approximately 60–90 ng/dl)[15] and in humans, this value is in the vicinity of 4 n*M* (approximately 120 ng/dl)[16].

A second mechanism is suggested by the study of Marks et al., in which hypogonadal men were treated with intramuscular injections of T every 2 weeks for 6 months[17]. Serum T increased substantially from baseline, as expected; however, intraprostatic concentrations of T and DHT (dihydrotestosterone) were not significantly different from baseline. A number of genetic markers of cellular proliferation were likewise unchanged[17]. This suggests that large changes in serum androgens are not reflected in the prostate, and raises a possibility that the prostate somehow achieves a relatively homeostatic microenvironment with regards to androgens, with optimal androgen concentrations achieved at low serum levels of T.

## ADDITIONAL EVIDENCE REGARDING T AND PCA

More than 20 English-language longitudinal studies have been published that investigated the relationship between endogenous serum T or other hormones, and the risk of development of PCa. In theory, if higher androgens truly caused more rapid PCa growth, then one should expect men with higher serum T to develop a greater rate of clinical PCa compared with men with lower serum T, given the high rate of occult PCa noted in men at autopsy studies. Yet, none of these studies revealed any significant relationship between total T and PCa, with a small minority reporting some association with minor androgens, ratios of hormones, or with isolated positive associations that were inconsistent with the remainder of results in the same study[5].

In 2008, a global collaborative effort pooled primary data from 18 of these longitudinal studies, comprising approximately 95% of the extant data on this topic[18]. With 3,886 men with PCa and over 6,438 men without cancer serving as age-matched controls, this study represents one of the largest PCa studies of any type, and was thus powered to detect even small variations in risk. Men were divided by quintiles according to serum concentrations of various sex hormones. The results were definitive: no relationship was seen between PCa risk and serum concentrations of T, free T, DHT, or other androgenic measures[18,19].

An additional area of interest is whether T therapy may pose a risk for PCa. Calof et al. performed a meta-analysis of 19 placebo-controlled T therapy trials in hypogonadal men[20]. No increased risk of PCa was noted in men treated with T vs. placebo, nor was there a higher rate of men who developed a PSA greater than 4.0 ng/ml. Interestingly, voiding symptoms also did not worsen in T-treated men vs. controls.

A somewhat surprising observation is that low serum T has been found in association with PCa in a number of studies[21]. For instance, men with increasingly severe reductions in serum T and PSA less than 4.0 ng/ml are at increased risk of positive prostate biopsy[22]. Similarly, a low ratio of T to PSA has also been shown to be a risk factor for the presence of PCa on biopsy[23]. In addition, low serum T has been found in association with high Gleason score[24,25], risk of biochemical recurrence following radical prostatectomy[26], stage at presentation[27], and survival[28].

## IMPLICATIONS OF THE SATURATION MODEL

There are important clinical ramifications of the Saturation Model, and the shift in concepts regarding the relationship of T and PCa. Ongoing research into the use of T therapy in T-deficient men continues to demonstrate a number of significant health benefits, not only in the traditional realm of sexual medicine, but also with regard to body composition, bone density, and mood[29]. Since the fear of PCa has been a major hurdle towards more frequent use of T therapy[30], it is likely that more physicians will consider use of T therapy for their patients if the concern regarding PCa is reduced.

A special population that has already benefited from a rethinking of the historical fears regarding T and PCa is men who have been treated for PCa with apparent cure. A large number of men in the U.S. and other countries have undergone curative treatment for localized PCa with little risk of cancer recurrence, and many of these men are symptomatically hypogonadal. Whereas a prior history of PCa has been considered an absolute contraindication to the use of T therapy for decades, there are now a series of reports that indicate low risk of disease progression or recurrence in men who have undergone definitive PCa treatment who subsequently were treated with T[31,32,33,34].

## DISCUSSION

The long-standing assumption that higher T necessarily causes enhanced growth of PCa in otherwise untreated men is a good example of an unexamined historical belief that has been proven to be false on closer investigation[5]. Although medical or surgical castration clearly causes PCa to regress, and restoration of normal T concentrations from castrate levels also clearly results in PCa growth, there appears to be a limit to the ability of androgens to stimulate PCa growth. Thus, men with high T appear to be at no greater risk of PCa development than men with low T[12,18] and studies have failed to show that high T predisposes men for worrisome outcomes once PCa has been diagnosed. In fact, a number of studies has shown a significant association of poor outcomes with low concentrations of T[21].

Where does the saturation point occur? This question is of considerable importance since men with T levels above this point should be at no greater risk of additional prostate growth with T therapy. Although this value has not been rigorously studied in men with PCa, studies reveal that maximal AR binding to androgen occurs at 60–90 ng/dl in the rat, and at approximately 120 ng/dl in benign human prostate tissue[12,15,16]. Since most T-deficient men have naturally occurring T concentrations above this point, this would explain why T administration causes only a minor increase in PSA, usually no greater than placebo-treated men. However, it must also be recognized that men who are severely T deficient who receive T therapy may indeed experience a substantial rise in PSA, with or without the presence of cancer. The Saturation Model explains why PCa occurs rarely in young men, despite the simultaneous presence of PCa microfoci and high T levels.

It is time to acknowledge that our traditional stance regarding T and PCa is inconsistent and illogical. For example, we allow serum T levels to rise within the normal range after discontinuation of LHRH agonist treatment with radiation therapy, yet we generally withhold T therapy from those who are symptomatic from failure of T levels to normalize, citing concerns of PCa progression. Although it has been widely believed that naturally occurring low levels of T are protective against development or progression of PCa, multiple studies have shown that low T offers no such protection[22,35], and may even be associated with increased PCa risk or worrisome cancer features when present.

Although there is yet to be a large, long-term controlled study on the effect of T therapy on PCa risk, substantial literature does exist that investigates the effects of androgens on prostate tissue. This literature, taken as a whole, presents a fairly benign picture regarding the PCa effects of T therapy. Even in men with known histories of PCa, there appears to be little risk that T therapy precipitates unexpected cancer recurrence[31,32,33,34]. If the risk of cancer progression or recurrence is low in such an at-risk population, how reasonable is it for clinicians to worry about the cancer risk of T therapy in the general population? The withholding of T therapy in men due to the fear of PCa risk or progression no longer is tenable in an age of evidence-based medicine, since neither evidence nor theory supports this position any longer.

## CONCLUSIONS

The longstanding belief that higher T leads to greater PCa growth in noncastrated men is contrary to a large body of accumulated evidence. The Saturation Model appears to resolve the dual observations that PCa growth is sensitive to changes in serum androgens at very low concentrations, but indifferent to such changes at concentration beyond the near-castrate range. Although there are no large, long-term studies on the safety of T therapy with regard to PCa, there is considerable evidence that PCa risk is not increased with higher serum T in noncastrated men. With proper medical monitoring, T therapy can be safely offered to men with T deficiency. The historical fear that T therapy will lead to greater PCa risk appears unfounded and should be discarded.
